# HSP70: a promising target for laryngeal carcinoma radiaotherapy by inhibiting cleavage and degradation of nucleolin

**DOI:** 10.1186/1756-9966-29-106

**Published:** 2010-08-06

**Authors:** Jing Xu, Kangkai Wang, Xin Zhang, Yuanzheng Qiu, Donghai Huang, Wei Li, Xianzhong Xiao, Yongquan Tian

**Affiliations:** 1 Department of Otolaryngology, Xiangya Hospital, Central South University, Changsha, PR China; 2 Department of Pathophysiology, Xiangya Hospital, Central South University, Changsha, PR China

## Abstract

Previous studies have shown that heat shock proteins (HSPs) were upregulated in various types of tumors and were associated with histological grade, recurrence and metastasis of malignant tumors. In this study, we investigated whether heat shock protein 70 kDa (HSP70) was associated with histological grade of laryngeal squamous cell carcinomas (LSCC). We also determine the role of HSP70 in LSCC radiation resistance using a laryngeal carcinoma xenograft model by antisense HSP70 RNA technique. Immunohistochemistry data showed that HSP70 was detected in 96% of LSCC tissues (48 out of 50). The expression level of HSP70 was significantly lower in early stage of LSCC than that in late stage (P = 0.015). Radiation treatment result showed that the volumes and weights of implantation tumors in the group injected with antisense HSP70 oligos were significantly reduced comparing to the group injected with random oligos(p < 0.05). In addition, cleavage and degradation of tumor nucleolin in antisense HSP70 oligos injection group was significantly higher than that in random oligos injection group. Our result suggested that HSP70 may play a role in LSCC radiotherapy resistance by inhibiting cleavage and degradation of nucleolin.

## Introduction

HSP70 is the most important member of heat shock protein family, and plays an important role in the cells endogenous protection mechanisms [1,2]. Recent studies have shown that different type of heat shock protein upregulated in different type of tumors, HSPs were associated with histological grade, recurrence and metastasis of malignant tumors [3-6]. However, the role of HSP70 in LSCC is not fully understood.

Weber, A *et al *had reported that HSP70 was significantly upregulated in squamous cell carcinoma of the head and neck (SCCHN) [7]. In our previous studies, we also found that HSP70 highly expressed at 7.7 folds comparing to normal tissue using HG-U133.Plus.2.0 chip (data unpublished). These results suggested that the overexpression of HSP70 was an important biological characteristic of LSCC.

Nucleolin(C23) is an abundant nuclear protein located in the dense fibrillar components(DFCs) and granular components(GCs) of nucleoli. It plays essential roles in promoting cell proliferation [8-11]. Our previous studies have shown that HSP70 could interact with C23 and inhibiting H_2_O_2_-induced cleavage and degradation of C23, thereby inhibiting reactive oxygen species-induced cell apoptosis [12]. There were two ways for radiotherapy to destruct tumor cells: (1) X-ray directly broke the DNA of the cancer cells into fragmentations, leading to cell apoptosis; (2) X-ray released free radicals from other components (e.g. H_2_O) in the cells thereby to attack tumor cells. Theoretically, radiotherapy could result in cleavage and degradation of C23 and sequentially kill the tumors.

In the present study, we determined whether reduction of HSP70 expression could enhance radiosensitivity of LSCC by increasing C23 cleavage and degradation.

## Materials and methods

### Tissue Microarray

High-quality tissue microarray (TMA) was constructed with fifty tumor samples including different stages of LSCC. The clinicopathologic features of the participants included in this analysis were presented in Table 1. Briefly, serial 5-μm sections were cut from each of the donor blocks. One of these sections was stained with hematoxylin and eosin staining (H&E) to mark morphologically representative areas of the tumor. Two areas in each case were targeted. Tissue cylinders with a diameter of 0.6 mm were punched from the two targeted areas in each donor block and deposited into a 14 × 7+2 (100 cores) TMA block, which contained 50 cores of tumor tissues. At last we gained 80 slides of high-quality TMA. Immunostaining for HSP70 protein was performed by using TMAs.

**Table 1:** Clinicopathologic characteristics of participants of TMA

Clinicopathologic characteristics of participants of TMA	
Male	45
Female	5
Average Age	61.3 ± 4.2
Stage I, II	21
Stage III, IV	29

### RNA oligos

According to the design principle of oligodexynucleotide (ODN) probes described by Myers KJ and Branch AD [13,14], three antisense-ODNs (ASODNs) were designed artificially against the HSP70 mRNA complete sequence (GeneBank NO.BC002453) from http://www.ncbi.nlm.nih.gov/. Three ASODNs were synthesized with phosphorothioate modification by Bioasia Co. Ltd. (Shanghai, China). After screening an effective ASODN, AS-1(5'-X TGTTTTCTTGGCCAT -3'), which complemented to the first 20 coding sequences of HSP70 mRNA, random oligos (5'-X GATTATCGTGTTGTTACT -3') were used as negative controls against AS-1, X represents green fluorescent marker.

### Animals and treatment

BALB/c female mice (18-22 g, 4-6 weeks) were obtained from Laboratory Animal Centre, Xiangya School of Medicine, Central South University (changsha, China). The animals were housed for 1 week prior to experiment. The animal experiments were undertaken within the guidelines of regulations for the use of experimental animals of Central South University. The animals were injected with 2 × 10^6 ^Hep-2 cells to establish the implantation tumor model of LSCC. When the implantation tumor grew up to 100 mm^3^, the nude mice were randomly divided into group antisense and group random. Each group has eight mice. Group antisense was injected with antisense oligos and group random was injected with random oligos. In all experiments, unless otherwise stated, the mice were administered with RNA oligos through intratumoral injection at the dose of 100 μg per 0.1 ml/injection at 7th, 10th and 14th day after tumor cells implantation. Three days after the final injection, all the mice accepted one single dose (5Gy) whole body radiation. The tumor volumes were measured twice a week using the formula: V = π/6 × (larger diameter) × (smaller diameter)^2 ^, as reported previously[15] . The mice were sacrificed once the tumor appeared necrosis, the tumor tissues were collected for western-blot, and paraffin-embedded tissues were used for immunohistochemistry and TUNEL assay.

### Western blot

The total protein was extracted from fresh tissues and the concentration of protein was determined by using bicinchoninic acid (BCA) Protein Assay Kit (Pierce, Rockford, U.S.A.). 100 μg of total protein was separated at 8% SDS-PAGE by electrophoresis and then transferred onto nitrocellulose membrane (Millipore, Bedford, U.S.A.). The membranes were blocked with 2% albumin in TBST (20 mM Tris-HCl, pH 8.0, 150 mM NaCl, 0.1% Tween-20) overnight at 4°C and then hybridized with the following primary antibodies: anti-HSP70 monoclonal antibody (Santa Cruz, USA), anti-nucleolin polyclonal antibody (Santa Cruz, USA), anti-β-actin (Boster Biological Technology, China). The immune complexes were visualized with DAB staining kit (Boster Biological Technology, China).

### Immunohistochemistry

4 μm tissue sections of implantation tumor samples were baked at 60°C overnight, deparaffinized in xylene and rehydrated through graded ethanol. Next, 3% hydrogen peroxide was applied to block the endogenous peroxidases for 30 minutes and sections were subjected to microwave heat-induced antigen retrieval in citrate buffer (0.01 M, pH 6.0) at high power for two times, each 7 minutes. After rinsing with phosphate-buffer saline, the sections were incubated with normal goat serum for 30 minutes at 37°C to block nonspecific binding. The samples were then incubated at 37°C for 30 minutes with mouse anti-HSP70 monoclonal antibody (Santa Cruz, USA) and the second antibody (rabbit anti-mouse antibody, MaiXin Bio, Fuzhou, China) for 30 minutes at 37°C. The streptavidin-biotin-peroxidase complex (SABC) tertiary system (MaiXin Bio) was used according to the manufacturer's instruction. All slides were visualized by applying 3,3- diaminobenzidine tetrahydrochloride (DAB) for 2 minutes and then counterstained with hematoxylin. The protein expression of HSP70 was thus determined as negative and positive. In addition, the expression levels of the HSP70 were also divided into low expression one (1+) and high expression one (2+ or 3+).

### TUNEL Assay

4 μm tissue sections of tumor samples were baked at 55°C for half an hour, deparaffinized in xylene and rehydrated through graded ethanol, and then incubated in the solution containing 1 μg/ml proteinase K/10 mM tris solution for 15 min at Room temperature. After rinsing with phosphate-buffer saline (PBS), the sections were incubated with TUNEL reaction mixture for 60 minutes at 37°C, and then were incubated in 100 μl anti-FITC-AP conj (converter-AP) for 30 min at 37°C. After incubation, the slides were covered with 50-100 μl substrate solution, incubated at room temperature, and visualized with DAB staining kit. The apoptosis cells were defined as negative and positive according to immunohistochemical staining. In addition, the rate of the apoptosis cells was also divided into low expression (1+) and high expression (2+ or 3+).

### Statistical Analysis

Data were represented as means ± S.E.M. of the number of independent experiment indicated (n) or experiments performed on at least three separate occasions. For cytoplasmic staining, the intensity of immunohistochemical staining was measured using a numerical scale (0 = no expression, 1+ = weak expression, 2+ = moderate expression, and 3+ = strong expression), and the statistic analysis for cytoplasmic staining was calculated using the Wilcoxon signed-rank test. A Student's t test was used to compare the volumes and weights of each group. All statistical analyses were performed by using the SPSS software package (version 10.0, Chicago, IL, USA). All tests were two-sided and P < 0.05 was considered statistically significant.

## Results

### HSP70 expression in different clinical stages of LSCC

**Figure 1:**
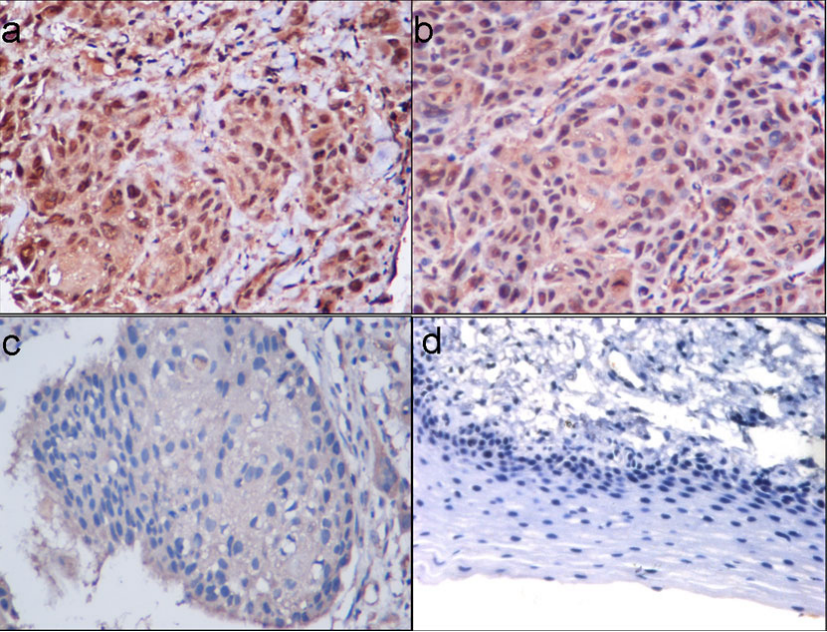
**HSP70 expression in LSCC tissues**. High HSP70 expression in late stage of LSCC tissues (a-b) was showed comparing to the low HSP70 expression in early stage LSCC(c), (d) is the negative control show the specificity of the antibody. HSP70 shows strong expression (scored as 3+, a), moderate expression (scored as 2+, b), weak expression (scored as 1+, c), and negative expression (scored as 0, d) in cytoplasm(×400).

To determine whether HSP70 was associated with histological grade of LSCC, we used tissue array to detect HSP70 expression in fifty LSCC cases including different stages. The results showed that staining of HSP70 was predominantly detected in cytoplasm as previously described [16]. The positive staining of HSP70 (Fig. 1a-b) was detected in 96% of LSCC tissues (48 out of 50). HSP70 was undetectable in 4% of LSCC specimens (Fig. 1c). The expression level of HSP70 and the clinical stage of LSCC were summarized in table 2. 19 out of 29 later stage patients have moderate expression, while 9 out 29 have strong expression, only 1 has weak expression. On the other hand, only 3 out 21 earlier stage patients have strong expression, 10 have moderate expression, the rest of the patients either have no expression or have weak expression. The data indicated that the expression levels of HSP70 protein in early stage cases were significantly lower than that in late stage cases (P = 0.015) (Wilcoxon signed-rank test).

**Table 2:** Analysis the HSP70 protein expression levels in early stage LSCC and than in late stage LSCC

		HSP70 expression levels				
			
Clinicopathological parameter	*n*	0	1+	2+	3+	*P*
stage I - II	21	2	6	10	3	
stage III - IV	29	0	1	19	9	**0.015**

### Screening the antisense oligodeoxynucleotides (ODNs) which could downregulate HSP70 expression in Hep-2 cells effectively

**Figure 2:**
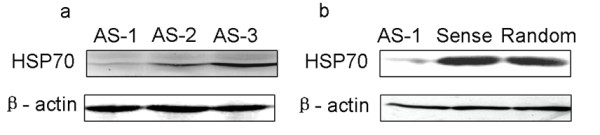
**Knock-down effect of HSP70 antisense oligos**. Hep-2 cells were transfected with HSP70 antisense oligos (AS) or control oligos (sense oligos and random oligos) as described under materials and methods. After incubation for 48 h, cells were harvested, lysed. Western-blotted (WB) with the corresponding antibodies was carried out to show the knock-down effect of AS. AS-1 has the best knock-down effect of all 3 oligos.

Based on the mRNA complete sequence of human HSP70 (GeneBank accession NO. BC002453), we designed three antisense oligos (ASODNs) at the different sites in human HSP70 sequence (AS-1, AS-2 and AS-3). After being transfected with HSP70 ASODN for 48 h, the total proteins were isolated and the expression level of HSP70 was determined by western blot. The results showed that AS-1 significantly inhibited the expression of HSP70. Both AS-2 and AS-3, however, did not show any effect (Fig 2a). All the following experiments were thus carried out by using AS-1. And the corresponding sense and random oligos were designed based on AS-1 sequence. Western blot showed that the random and sense oligos had no repressive effect on the expression of HSP70 (Fig 2b).

### The radiation sensitizing effect of HSP70 antisense oligos on laryngeal carcinoma

**Figure 3:**
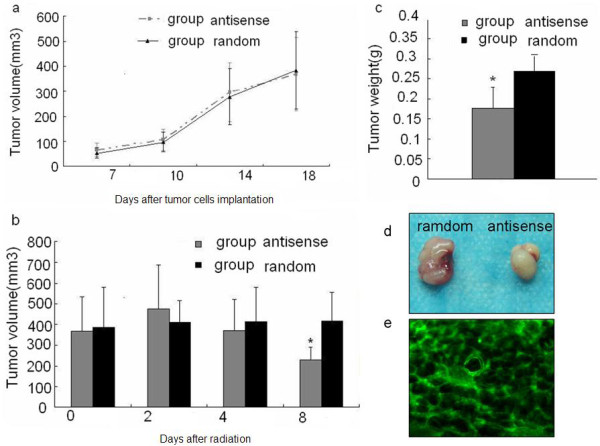
**Effect of HSP70 antisense oligos on radiotherapy of laryngeal carcinoma xenografts**. (a) shows the tumor growth curve before radiation, no difference between the 2 groups were found, the tumor volum were for antisense and random group, respectively (P > 0.05, 368 ± 129 mm3vs 384 ± 179 mm3). (b)There was significant difference in tumor volumes after 5Gy radiation (P < 0.05, 229 ± 28 mm3 vs 417 ± 103 mm3) (c) Tumor weights also showed significant difference after 5Gy radiation (P < 0.05, 0.18 ± 0.04 g vs 0.27 ± 0.05 g). (d) showed the representative sample of group antisense and group random after 5Gy radiation (e) showed the infection efficiency of intratumoral injection.:100×. n = 8 per group,* < 0.05.

To investigate whether the HSP70 antisense oligos have radiation sensitizing effect on laryngeal carcinoma xenografts in vivo, the antisense and random oligos were injected into tumor through intratumoral injection. The mice were treated with radiation (5Gy). The treatment effect was measured. The results showed that there was no significant difference in the tumor growth between group antisense (368 ± 129 mm3) and group random(384 ± 179 mm3) before radiotherapy (P > 0.05, Fig. 3a). However, eight days after radiotherapy, the volumes and weights of implantation tumor in group antisense (229 ± 28 mm3 and 0.18 ± 0.04 g) were significantly smaller than that of group random (417 ± 103 mm3 and 0.27 ± 0.05 g) (P < 0.05; Fig. 3b, c, d). To determine the efficiency of intratumoral injection, we used oligos with green fluorescent marker and observed the tumor under a fluorescence microscope after the first injection. Fig. 3e shows obvious infection efficiency.

### HSP70 antisense oligos downregulated the HSP70 expression in laryngeal carcinoma xenografts

**Figure 4:**
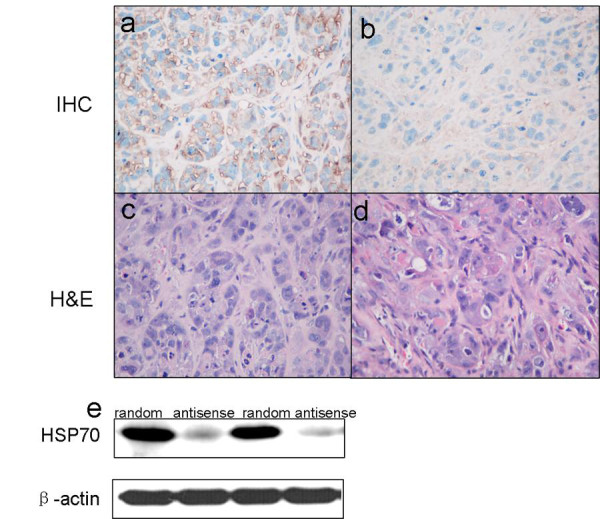
**HSP70 expressions in laryngeal carcinoma xenograft were down-regulated by HSP70 antisense oligos**. (a) shows HSP70 expression in implantation tumor treated with random oligos. (b) shows HSP70 expression in implantation tumor treated with HSP70 antisense oligos. (c-d) shows the representative H&E images in group random negative controls and group antisense; Western blot shows hsp70 expressions in group antisense and group random (e). HSP70 expression is significantly reduced in the antisense group comparing with random group.

To further determine the inhibitory effect of HSP70 antisense oligos on HSP70 expression, HSP70 in each group was detected by western blot (4e) and immunohistochemical staining (Fig. 4a, b). The results showed that HSP70 antisense oligos significantly downregulated HSP70 expression in laryngeal carcinoma xenografts as it is shown in both western-blot and immunohistochemistry assay.

### Cleavage and degradation of C23 by HSP70 antisense oligos promoted radiation-induced apoptosis

**Figure 5:**
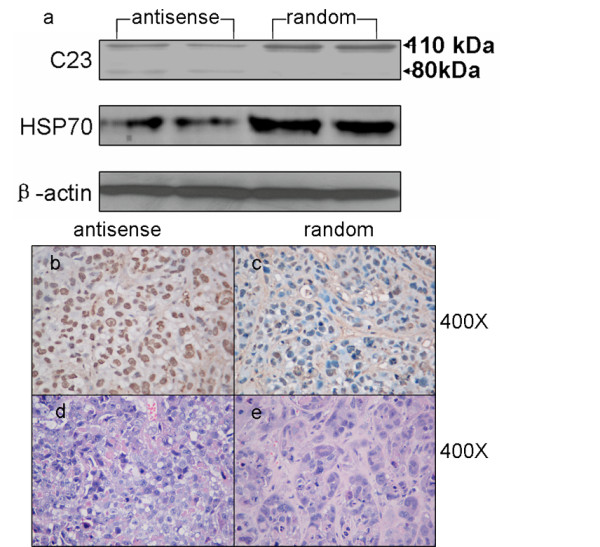
**Expression levels of HSP70 and cleavage and down-regulation of C23**. (a) Western blot detected HSP70 and C23 expression in group antisense and group random; (b-c) the representative images of TUNEL assay in group antisense and group random; (d-e) The representative H&E images in group antisense and group random negative controls (×400).

The levels of cleavage and degradation of C23 in each group were detected by western blot. The results showed that in the random group, a major immuno-positive band with an estimated molecular weight of 110-kDa was observed while the staining intensity of the 110-kDa band was decreased in the antisense group (Fig 5a). Moreover, an 80 kDa cleaved band of C23 was detected in the antisense group while this 80-kDa band was not detected in the random group (Fig 5a). These results indicated that HSP70 down-regulation was associated with cleavage and degradation of C23. Moreover, the apoptosis cells in each group were identified by TUTEL method. The results showed that more apoptosis cells in group antisense were observed than that in group random (Fig. 5b, c, d, e). This result suggested that HSP70 reduction were associated with cleavage and degradation of C23 and tumor cell apoptosis.

## Discussion

As one of the most conserved molecular chaperones, HSP70 is essential for proper folding and assembly of proteins^1,2^. It has been reported that HSP70.1 and HSP70.3 gene play a key role in the maintenance and repair of chromosome stability in mouse fibroblast cell after radiation, the radiosensitivity in HSP gene knockout mice is higher than that in wild-type mice [16]. This indicates HSP70 is an important radiation-resistance gene. However, this result came from the non-tumor cell experiment. Herein, we used Hep-2 cell line, which has a high expression level of endogenous HSP70 protein, to establish a laryngeal carcinoma xenograft model. The HSP70 antisense oligos was used to block HSP70 expression. Our results showed that HSP70 antisense oligos treatment increased radiation sensitizing activity in xenograft tumors. These results suggested that HSP70 may play an important role as a radiotherapy-resistant gene in laryngeal carcinoma.

It has been shown HSP70 could interact with nucleolin (C23) and inhibit H_2_O_2_-induced cleavage and degradation of C23 [10]. C23, a nonhistone nuclear RNA binding protein, plays an important role in maintaining the balance between anti-apoptosis and pro-apoptosis [8,9]. Our study has shown that blocking HSP70 expression could promote cleavage and degradation the expression of C23 on laryngeal carcinoma xenograft after radiotherapy. Nucleolin was cleaved and degraded during several apoptotic cell models. Previous studies have showed radiotherapy could induce a typical apoptotic cell death by breaking nucleolin into fragmentations [17,18]. Western-blot results of the cleavage and degradation of nucleolin showed that a cleaved band (80 kDa) of nucleolin appeared after radiotherapy by a single dose of 5Gy. Cleavage and degradation of nucleolin was also observed in both group antisense and group random which indicated that cleavage and degradation of nucleolin was a typical response to laryngeal carcinoma xenograft damage caused by the radiotherapy. The over-expression of HSP70 inhibited cleavage and degradation of nucleolin, and induced radiotherapy resistance.

Taken together, our data suggested that cleavage and degradation of nucleolin were involved in the apoptosis induced by radiotherapy, HSP70 serve as an radiotherapy resistance gene by inhibiting the cleavage and degradation of nucleolin.

Since the complex nature of the mechanisms in apoptosis and the multi-functionality of HSP70, there are still several questions remain to be answered inorder to address the role of HSP70 in radiation resistance. One interesting question is which domain of HSP70 is involved in the cleavage and degradation of nucleolin. It will also be interesting to know if nucleolin plays an essential role in radiation induced apoptosis. A nucleolin overexpression and knock-out model will be highly valuable to address this issue. The role of each HSP70 functional domain in protecting C23 are still yet to be determined.

## Competing interests

The authors declare that they have no competing interests.

## Authors' contributions

JX is responsible for experiment design and perform as well as data analysis. KW is designed the anti-sense oligos. XZ is responsible for data analysis guide. DH is responsible for IHC staining. YQ, and XZ participate design and coordination of the experiment. YQ is responsible for designing the experiment and writing the paper. All authors read and approved the final manuscript.

## Consent

Prior consent was obtained from the patients for collection of laryngopharyngeal specimens in accordance with the guidelines of Xiangya Hospital; the current study protocol was approved by the ethical committee at Xiangya Hospital of Central South University.
